# Treatment of gingival class I or class II recession using subepithelial connective tissue graft and acellular dermal matrix allograft

**DOI:** 10.6026/97320630018801

**Published:** 2022-09-30

**Authors:** Ridhima Sood, Sumanpreet Shergill, Jyotsana Singh, Ena Sharma, Garg Ridhi

**Affiliations:** 1 Rayat Bahra Dental College, Mohali, Punjab, India

**Keywords:** Gingival recession, acellular dermal matrix allograft, subepithelial connective tissue graft, recession height, clinical attachment level

## Abstract

The present randomized control was conducted clinically to evaluate the effectiveness of Acellular Dermal Matrix Allograft (ADMA) and Subepithelial Connective Tissue Graft (SCTG) in combination with Coronally Positioned Flap (CPF) in the treatment of Miller's class I and II multiple gingival recession in aesthetic areas. A total of 20 patients aged between 18 to 40 years were selected for this study, meeting all the criteria for inclusion. 10 patients were treated with ADMA and 10 patients with SCTG in combination with CPF. Various clinical parameters were assessed viz. probing pocket depth (PPD), clinical attachment level (CAL), gingival recession height (RH) and width of keratinized gingiva (WKG) at baseline and 6 months after surgery. The mean RH at baseline in the control and test groups was 3.05 ±0 .55(mean± SD) and 2.60 ±.99 respectively. At 3 months the mean RH was found to be 1.60±0.74 and 1.05 ± .60 in the control and test group respectively. The mean percentage of root coverage (MRC%) at 6 months in the control and test group was 65.69 ±26.52 (mean± SD) and 65.54 ±.9.16 respectively but no statistically significant difference was seen between the two groups. The results of the study suggest that the combination of both subepithelial connective tissue graft and acellular dermal matrix graft with a coronally positioned flap can produce an equivalent amount of esthetic root coverage.

## Background:

The progression of recession defects warrants an investigation of the etiologic factors and their elimination. The consideration of therapeutic actions is directed at minimizing the apical movement of the gingival margins. At the same time, achieving predictable and esthetic root coverage is the main objective of periodontal plastic surgery. The subepithelial connective tissue graft (SCTG) has been viewed as an effective and predictable method of achieving the coverage of denuded root surface in Miller's class I and class II gingival recession. Langer and Calagna in 1980 gave the technique of subepithelial connective tissue graft (SCTG) and later it was modified in 1985 by Langer and Langer. Since then various modifications have been made in the incisions and designs of this technique to make it a treatment of choice for achieving predictable root coverage and aesthetics. [1,2] The subepithelial connective tissue graft is considered as a gold standard 2 in the treatment of gingival recession due to its high predictability for root coverage and increase in the width of keratinized tissue. However, the main limitation of this technique is the need for a second surgical site. Besides this, the donor tissue from the palatal region may be insufficient for treatment of multiple recession sites and the patient may be subjected to multiple surgeries just to harvest the donor graft tissue. [2] These limitations led to the development of newer techniques that can achieve predictable root coverage with good esthetics. Recently, the use of an ADMG called Alloderm® has become an increasingly popular technique as a substitute for connective tissue graft. [3,4] Alloderm is obtained from a human donor skin tissue, processed that removes its cell component while preserving the remaining bioactive components, which are subsequently freeze-dried. The preparation of this dermal allograft involves cell component removal and preservation of the ultra structural integrity, which if damaged would induce an inflammatory response. [5-7] ADM was originally utilized for use in plastic surgery for the treatment of full-thickness burn wounds. [8] A periodontal plastic surgical procedure using ADM offers the advantage of avoiding the need for a second surgical palatal donor site thereby offering the clinician the tissue thickness similar to that of an autogenous connective tissue graft. [9, 10] Therefore, it is of interest to evaluate the effectiveness of ADMA and SCTG in combination with coronally advanced flap in the treatment of Miller's class I or class II gingival recession.

## Materials & Methods:

Twenty systemically healthy patients aged between 18 to 40 years were selected from the Outpatient, Department of Periodontics, I.T.S centre for dental studies and research. The following patients were included in the study that were systemically healthy, non smokers and had Miller's Class I or II recession defects on maxillary incisors, canines or premolars. Exclusion Criteria were as follows supra-erupted tooth, non-vital tooth, subjects who have taken antibiotics in the past 3 months, are on immunosuppressant drugs or any medication known to cause gingival enlargement, with active infectious diseases (hepatitis, tuberculosis, HIV infection), with active caries or restoration on the root surface of the concerned tooth, pregnant and lactating mothers, high frenal attachment and who have previously undergone any type of periodontal surgical procedure or regenerative therapy in the past one year. The surgeries were performed from October 2013 to January 2014 (IIEC NO(ITSCDSR/IIEC/2012-15/PERIO/001).

### Standardized clinical parameters:

The following clinical parameters were measured for assessment of the results in all the selected cases: plaque index (PI), [11] Gingival index (GI), [12] probing pocket depth (PPD), clinical attachment level (CAL), recession height (RH) and width of keratinized gingiva (WKG) [13] by using UNC-15 periodontal probe. All the probing measurements were recorded (Mid-buccally per tooth) at baseline, 3 and 6 months. The clinical parameters were measured using a UNC-15 probe. Reproducible alignments of the probe were provided by custom-made self-cure acrylic stents grooved in an occluso-apical direction corresponding to the inter-proximal area. [14].

### Pre-surgical management:

Prior to the surgery, all the patients received thorough supra gingival and subgingival scaling and root planing and oral hygiene instructions were given to the patient. Patients were evaluated for optimal oral hygiene at the end of 2 weeks. Every effort was made to modify the habits that contributed to the recession defects. Occlusal analysis was within the normal limits in all the cases included in the study and no occlusal therapy was performed in any case.

### Surgical procedure:

After local anesthesia, the exposed root surface was planed with hand and ultrasonic instruments. The recipient site was prepared by giving an intra-sulcular incision at the labial aspect of the involved teeth. Two horizontal papilla sparing incisions were made at right angles to the adjacent interdental papillae at the level of CEJ without interfering with the gingival margin of the neighbouring teeth. Two oblique vertical incisions were extended, beyond the muco gingival junction and the muco periosteal flap was raised, up to the muco gingival junction. After this point, a split-thickness flap was extended apically, releasing the tension and favouring the coronal positioning of the flap. [15] The epithelium on the adjacent papillae was de-epithelised. The exposed root surfaces were planed with Gracey curettes (3/4) and conditioned with 24% EDTA gel for 2 minutes to remove the smear layer followed by subsequent rinsing with sterile saline solution to obtain a surface devoid of organic debris and gently air-dried. SCTG in a proper dimension was harvested from the palate by trap door technique and trimmed as necessary. [16] (The graft was placed at the CEJ level and 2-3mm beyond to margin of alveolar bone covering the entire defect and stabilized by using 4-0 resorbable sutures and then sutured to the neighbouring mucosa. Flap was then coronally positioned with margins located on the coronal to the cement-enamel junction. It was secured in position horizontal suspensory sutures through an orthodontic bracket placed at the mid- buccal portion of the crown. [17] Additional lateral interrupted sutures were placed to close the wound of the releasing incisions. In the test group ADMA to be adapted after being aseptically rehydrated in sterile saline according to the manufacturer's instruction. The graft was trimmed coronally so that it was at CEJ and apically it covered the alveolar bone up to at least 2 to 3 mm. The connective tissue side was placed adjacent to the bone and the basement membrane side was placed facing the flap. [18] The borders of ADMA basically coronal and lateral sides were sutured to lingual gingival tissue with resorbable sutures. The flap was then coronally positioned and sutured to cover the ADMA. The periodontal dressing was placed over the surgical site in both groups.

### Postoperative care:

Postoperative medications included a single standard regimen of oral administration of Amoxicillin 500 mg thrice daily for 5 days, and Ibuprofen 400mg + Paracetamol 325mg thrice daily for 5 days, along with 10 ml of 0.2% chlorhexidine gluconate rinse twice daily for a period of 2 weeks. The patients were asked to resume mechanical tooth brushing one month after the treated area using the roll technique with a soft toothbrush [19]. Patients were called after 2 weeks for periodontal dressing and suture removal.

### Statistical analysis:

The software used for the statistical analysis are SPSS (statistical package for social sciences) version 21.0 and Epi-info version 3.0. The values were represented in Number (n), Percentage (%) and Mean (U). The statistical tests used were Unpaired or independent samples t-test is used for comparison of mean value between test and control groups (Inter group comparison) A one way ANOVA (Analysis of Variance) test, post hoc – Bonferroni multiple test and paired t–test was used to evaluate the statistical significance of differences at different time intervals (baseline, 3 months and 6 months) respectively. The p-value was taken significantly when less than 0.05 (p<0.05) and a confidence interval of 95% was taken.

## Results

In the present study, 20 patients(18 males and 2 females) with ages ranging from 20-45 years (mean age 29.75±4.35), fulfilling the inclusion and exclusion criteria contributing to a total of 20 recession defects were recruited. The recession defects were located in 9 canines, 4 premolars, 2 central and 5 lateral incisors. The mean CAL at baseline in the control and test groups were 4.80±0 .63 (mean± SD) and 4.00 ±1.20 respectively. The mean CAL at 6 months for the control group was 2.12±0.91 and 1.93±.84 for the test group respectively. The independent t-test (inter group comparison) was used to compare the mean CAL between the groups and the difference between the groups was found to be statistically non-significant at baseline, 3 months and 6 months respectively (Table 1, 2 - see PDF). The post hoc comparison(inter group comparison) of the scores showed that the mean CAL reduced significantly (p<0.05) from baseline to 3 months and baseline to 6 months respectively in both the control as well as test groups but no statistically significant difference was seen at 3 months to 6 months in both the groups (Table 3 - see PDF). The approximate size of recession defects in both the groups was nearly similar and there was no statistically significant difference in the mean preoperative gingival RH. The mean RH at baseline in the control and test groups was 3.05 ±0.55 (mean± SD) and 2.60 ±.99 respectively. The mean RH at 6 months for the control group was 1.14 ± 1.00 and 0.90±0.46 for the test group respectively (Table 1, 2 - see PDF). The post hoc comparison of the scores showed that the mean RH from baseline to 3 months and baseline to 6 months reduced significantly (p<0.05) in both the control as well as test groups but no statistically significant difference was seen at 3 months to 6 months in both the groups (Table 3 - see PDF). The mean WKG scores at baseline in the control and test group was 3 .80±0 .63 (mean± SD) and 4.05 ±.86 respectively. The mean WKG at 6 months for the control group was 4.45±0.50 and 4.80±0.59 for the test group (Table 1, 2 - see PDF). On intra group comparison at 3- time point intervals, the mean WKG reduced significantly at 3 months and 6 months. The post hoc comparison of the scores showed that the mean WKG reduced significantly (p<0.05) from baseline to 3 months and baseline to 6 months in the control group but showed a statistically non-significant difference from 3 months to 6 months.(p>0.05) The test group showed a statistically non-significant difference at baseline to 3 months and 3 months to 6 months (p>0.05) but a statistically significant difference at baseline to 6 months (Table 3 - see PDF). The mean percentage of root coverage (MRC%) at 6 months in the control and test groups was 65.69 ±26.52 (mean± SD) and 65.54 ±.9.16 respectively but no statistically significant difference was seen between the two groups (Table 4 - see PDF). Two of the sites in the control group showed 90% of root coverage, four of the sites showed 83.3% of root coverage at 6 months whereas one of the sites showed 66.7% of root coverage. The test group showed 80% of root coverage at 2 sites and 8 sites showed 66.7% of root coverage at 6 months. The patients were satisfied with the root coverage and colour match in both the treatment groups but minimal discomfort was reported by the patients in the test group when compared with the control group because of the postoperative morbidity due to the involvement of the second surgical site.

## Discussion:

The most common problem encountered by a clinician in his day to day practice is exposed root surface or a tooth getting long commonly reported by the patient. So the clinicians are faced with the challenge of not only addressing the biological and functional problems present in the periodontium but also fulfilling the main indication for root coverage procedures which is the esthetics and/or cosmetic demands followed by the management of root hypersensitivity, root caries or when it hampers proper plaque removal. [20] The purpose of this present randomized controlled clinical trial was to compare the clinical outcomes of traditional SCTG versus ADMA in combination with coronally advanced flap for the treatment of isolated Miller's Class I and Class II gingival recession. There was no sign of allergy, infection or any other complications during the study and all the patients tolerated the procedures well. The reduction in the plaque index and gingival index remained constant throughout the study period. In the present study, when the teeth were compared to the adjacent non-treated teeth there was no visual detectable increase in plaque accumulation. This suggested that patients were well motivated to maintain good oral hygiene postoperatively. This finding is in agreement with Chen et al. in 1995. [21] There was a slightly greater reduction in the probing depth in the control group when compared with the test group. The mean difference between the groups from baseline to 6 months was also statistically significant. However, within the control group and the test group, there was a statistically significant decrease in the probing depth scores from baseline to 3 months and 6 months. These results are in agreement with the study conducted by Harris reported a mean reduction of 1.2 mm in the control group and 0.7 mm in the test group respectively. The mean reduction in the recession height in both the control (1.91) and the test group (1.70) was nearly the same. Both the groups showed a statistically significant decrease in the recession height at baseline to 3 months and 6 months time intervals. In the present study, the reduction in the recession height in the test group could be attributed to the coronal movement of the gingival margin on the denuded roots following tissue grafts. There is a creeping attachment formation in one month after the graft placement has been referred in several studies. Harris referred to 0.85 mm creeping attachment through SCTG following one year and Piniprato et al. [22] referred to 0.43 mm through the coronally advanced flap. In the present study, both groups showed an increase in the width of keratinized gingiva. But the ADMA group showed a slightly greater increase in width of keratinized gingiva (4.80 ± 0.59), as compared to the SCTG group (4.50 ± 0.58mm) at 6 months. Both the groups showed a statistically significant increase in the width of keratinized gingiva at 3 and 6 months time intervals. However, the mean difference in the width of keratinized gingiva between the groups at baseline to 6 months was statistically not significant. Tal et al. [10] reported an increase of 2.0 mm in keratinized tissue when the basement membrane side of the acellular dermal matrix graft was placed facing the flap's connective tissue. In the present study, the basement membrane side of ADM was placed towards the flap so this could attribute to the slight increase in the width of keratinized gingiva in the test site when compared with the control site. One feature of this material is that it has 2 surfaces: one has characteristics of the basement membrane and the other of the connective tissue with collagen and elastic fibers. This non-inert structure acts as a biologically compatible framework into which fibroblasts, keratinocytes and basically epithelial cells can migrate and adhere hence repopulating and incorporating the material into the newly formed tissue. [23] The control group showed a greater CAL gain when compared with the test group. Both the groups showed a statistically significant increase in the clinical attachment level at 3 and 6 months time intervals. However the mean difference in the CAL gain between the groups at baseline to 6 months was statistically not significant. This finding is in accordance with the results obtained in studies as per conducted by Novaes et al. [24] and Paolantonio et al. [25] who reported no statistically significant difference in the CAL gain between the SCTG and ADMA groups. Harris et al stated that clinical gain in the attachment level may be due to a combination of new connective tissue attachment in the apical half of the defect and the presence of long junctional epithelial attachment in the coronal half. [26] The mean percentage of root coverage was the same in both groups. But there was no statistically significant difference seen between the two groups indicating that both the groups were found equally effective in the treatment of gingival recession and achieving adequate root coverage as well as esthetics. The results in the control group are in accordance as per the study conducted by Bouchard et al in which he reported a mean root coverage of 69.2% in the subepithelial connective tissue group. [27,29] In the present study the results in the test group are nearly value similar to the study conducted by Aichelmann-Reidy et al. who also reported a mean percentage of root coverage 65.9% with acellular dermal matrix graft. [28]

## Conclusions:

The SCTG is a versatile technique in which a bi-laminar vascular environment is created to nourish the graft. But harvesting the palatal graft increases postoperative morbidity and is time-consuming. In the current era that focuses on minimally invasive techniques, the use of allografts contributed to a significant reduction in patient morbidity and surgical risks. It provides an unlimited supply of graft material thereby permitting multiple site root coverage which can be extended for a sextant, quadrant, or even a full mouth arch at one time. The results provided by both the groups SCTG as well as ADMG were nearly equivalent and equally effective thereby suggesting that both the treatment modalities are feasible options for esthetic root coverage. However long-term clinical trials with a larger sample size are recommended to evaluate the stability of the root coverage procedures.
